# *In vivo* confocal microscopic study of cornea verticillata and limbus deposits in patients with Fabry disease

**DOI:** 10.3389/fmed.2025.1541510

**Published:** 2025-02-05

**Authors:** Xuecong Zhou, Yawen Zhao, Yingsi Li, Yujing Yuan, Xiaoming Yan, Wei Zhang, Yuan Wu

**Affiliations:** ^1^Department of Ophthalmology, Peking University First Hospital, Peking University, Beijing, China; ^2^Department of Neurology, Peking University First Hospital, Peking University, Beijing, China

**Keywords:** Fabry disease, *in vivo* confocal microscopy, cornea verticillata, epithelial deposits, limbus

## Abstract

**Purpose:**

This study was aimed to investigate the microstructure characteristics of cornea verticillata and limbus deposits in patients with Fabry disease (FD) using *in vivo* confocal microscopy (IVCM).

**Methods:**

A total of 60 eyes from 30 patients diagnosed with FD were examined and compared with 36 eyes from 18 healthy controls in this prospective, cross-sectional, controlled, single-center study. The initial assessment of cornea verticillata (CV) was conducted using slit-lamp microscopy. Subsequently, IVCM was performed to assess deposits in the corneal and limbal epithelium. We compared the differences between the sexes (heterozygous and hemizygous) and phenotypes (classical and non-classical).

**Results:**

The epithelial deposit detection rate with IVCM was statistically higher (52/60, 86.67%) compared to the biomicroscopic evaluation of CV using a silt lamp (46/60, 76.67%) (*p* = 0.031). A higher prevalence of corneal epithelial deposits was observed in the classical phenotype as compared to the non-classical phenotype (*p* = 0.023). Surprisingly, cardiac variants previously lacking cornea verticillata show a high prevalence (85.71%) of corneal epithelial deposits under IVCM. The prevalence and severity of deposits, especially in limbal epithelial rete pegs, were higher in FD than in controls (*p* < 0.001).

**Conclusion:**

Compared with slit-lamp microscopy, IVCM provides a more effective tool for examining the epithelial deposits in patients with FD. Patients with FD demonstrated a profound bilateral increase in corneal epithelial deposits and limbal hyperreflective cells compared to controls, with more prominent pathological changes observed in classical phenotype individuals. The high prevalence of epithelial deposits observed through IVCM in the cardiac variant highlights the essential ability of IVCM as an effective diagnostic tool.

## 1 Introduction

Fabry disease (FD) is an X-linked lysosomal storage disorder (LSD) caused by the mutations of the *α*-galactosidase (GLA) gene, leading to decreased or deficient activity of the lysosomal enzyme *α*-galactosidase A (α-Gal) (1–3). This enzymatic deficiency results in the progressive accumulation of glycosphingolipids, predominantly globotriaosylceramide (Gb3) and globotriaosylsphingosine (lyso-Gb3), in the plasma and lysosomes throughout the body. This accumulation leads to a variety of complex clinical manifestations that differ between the sexes (hemizygous males and heterozygous females) and phenotypes (classical and non-classical phenotypes) (2, 4–8). Due to more GLA variants being discovered and newborn gene screening technology being applied, the estimated prevalence of FD has increased from the previous 1/170,000–1/40,000 to the latest 1:1,250 (2, 9, 10). However, as a rare disease, misdiagnoses and delayed diagnosis of FD remain as significant issues. It is estimated that the median duration from the onset of initial symptoms to the final diagnosis of FD is 21 years, with the average age of final diagnoses being 41.1 years; this prolonged timeline contributes to the worsening, irreversible damage of the multisystemic disorders (11).

As one of the earliest clinical manifestations typically emerging during the second decade of life, ophthalmologic manifestations have evolved into one of the significant hallmarks of FD (12–16). The ocular changes provide ophthalmologists with a great opportunity to diagnose FD. However, apart from half of the diagnoses contributed by affected relatives, the medical specialists who most often establish the diagnosis of FD are nephrologists, neurologists, geneticists, and dermatologists, with ophthalmologists accounting for a small portion (17). Improving the recognition incidence of characteristic ocular changes by ophthalmologists is imperative in the early diagnosis of FD.

The cornea verticillata (CV) is one of the most common and characteristic ophthalmologic manifestations of FD, which mainly appears at the inferior part of the cornea bilaterally in the epithelial and subepithelial layer, manifested as whorl-like opacities (12–16, 18, 19). However, the prevalence of CV by slit-lamp microscopy examination results has fluctuated across different studies ranging from 14 to 95% in males and from 31 to 100% in females, which may largely depend on the physician’s level of experience (12, 13, 18, 20–25). A thorough and comprehensive examination of the cornea is needed to better understand corneal involvement in FD.

As a rapid, valid, non-invasive technique to observe detailed corneal structures, *in vivo* confocal microscopy (IVCM) enables identification of the corneal deposits in patients with FD (21, 26, 27). This advanced technology not only enables us to detect the distribution of Gb3 particles in the cornea but also allows us to quantitatively classify the degree of corneal deposits, which has significantly improved the detectable rate of CV (28). In addition, the hypothesized cause of the peculiar whorl pattern of CV is the centripetal movement of opacities in the flow of limbal epithelial stem cells migrating to the central cornea in previous studies (29–32). Real-time quantitative analysis of the cornea and limbus at the cellular level through IVCM enables a better understanding of the epithelial deposits.

This study aimed to determine a reliable evaluation of the cornea verticillata and limbus deposits using IVCM and to compare the extent of deposits among patients of between the sexes and phenotypes of FD. Better detection of corneal changes could enable earlier, more precise, and less invasive diagnosis in patients with FD, providing opportunities to receive earlier intervention and get a better prognosis.

## 2 Materials and methods

### 2.1 Patients

This prospective, cross-sectional, controlled, single-center study was conducted at the Department of Ophthalmology of Peking University First Hospital from June 2023 to May 2024. This study was approved by the Institutional Review Board of Peking University First Hospital (2022-364-002) and was conducted in accordance with the tenets of the Declaration of Helsinki. Informed consent was obtained from all participants.

This study included a total of 60 eyes from 30 patients with FD and 36 eyes from 18 healthy individuals. The confirmation of the diagnosis of FD was provided by the neurologists at our hospital, based on clinical history together with laboratory tests (GLA gene combined with *α*-Gal A activity, and lyso-Gb3) according to the Chinese consensus on FD (33). The systemic condition of patients with FD was screened by the neurologists through inquest of the case history and family history, physical examination, cardiac examination (electrocardiogram, echocardiography, or magnetic resonance imaging (MRI)), renal functions test (kidney puncture biopsy if necessary), GLA gene detection combined with *α*-Gal A activity and lyso-Gb3. Patients were classified as classical or non-classical phenotype based on their enzyme activity (hemizygous males only) and the presence or absence of characteristic manifestations according to Chinese consensus on FD (33) and previous studies (4, 34, 35). The classical phenotype consists of patients exhibiting significantly reduced or absent enzyme activity (≤5%) in hemizygous males, alongside a GLA mutation and one or more characteristic FD symptoms, including neuropathic pain, hypohidrosis, cornea verticillata, and/or angiokeratoma (4, 33). In one female patient (P10), the examination of enzyme activity and concentration of substrate was not performed at the time—she was classified as classical FD according to her GLA mutation and several characteristic FD symptoms (Supplementary Table S1). The patients who did not meet the criteria of the classic phenotype were classified into non-classical phenotype, among whom cardiac variants have cardiac symptoms (e.g., angina, arrhythmias, dyspnea, etc.), electrocardiogram, echocardiography, or magnetic resonance imaging (MRI) abnormalities [e.g., dysrhythmias, left ventricular hypertrophy (LVH), hypertrophic cardiomyopathy (HCM), myocardial fibrosis, etc.], and/or microalbuminuria (33, 34), while renal variants have proteinuria and renal failure (33, 35).

Patients with infectious keratitis and conjunctivitis, corneal surgery, corneal contact lens wearing history, eye continuous medication history, particular drug use (amiodarone and chlorpromazine), systemic diseases other than FD (autoimmunity disease and diabetes) were excluded from this study.

Each subject underwent double-blind examinations, with slit-lamp microscopy by one ophthalmologist to assess the presence of CV and graded it, followed by an *in vivo* confocal microscopy examination performed by an expert examiner.

### 2.2 Slit-lamp microscopy

Haag-Streit BQ-900 Slit Lamp with EyeCap v7.x Imaging System (Haag-Streit Diagnostics, Koeniz, Switzerland) was used to evaluate and photograph the cornea of all participants. The severity of CV based on the slit-lamp microscopy examination was classified into four grades, according to the grading system of amiodarone keratopathy proposed by Orlando et al. (36). In Grade 1 CV, round epithelial microdeposits are present at the inferior pupillary margin in the midperiphery. Grade 2 CV is characterized by linear filament-like deposits resembling cat’s whiskers, distributed beyond the inferior pupillary margin to the corneal limbus, while retaining a clear zone. With the increase and expansion of branches, a whorled pattern extending into the pupillary axis is formed in Grade 3 CV. In Grade 4, CV is characterized by whorled branching patterns accompanied by irregular clumps of deposits (36). The patient’s grading level is assigned as a weighted score, that is, a grade of 1 corresponds to a score of 1, a grade of 4 corresponds to a score of 4, and so forth.

### 2.3 *In vivo* confocal microscopy

#### 2.3.1 Image acquisition

The Rostock Cornea Module of the Heidelberg Retina Tomograph-III (Heidelberg Engineering, Heidelberg, Germany) was used to obtain the IVCM images of the cornea in all subjects. The microscope uses a 670-nm diode laser as the illumination source. A 63× water immersion objective lens contacted with the cornea through a disposable sterile corneal cap (TomoCap, Heidelberg Engineering, Heidelberg, Germany) and a layer of ophthalmic gel (Vidisic, Bausch & Lomb, Berlin, Germany). After anesthetized with 0.4% oxybuprocaine hydrochloride (Benoxi, Santen Pharmaceutical Co. Ltd., Osaka, Japan) instilled in both eyes, the subjects were positioned on a chin rest and forehead support, with the examined eye opened by an eye speculum and the fellow eye instructed to fixate on a target light for stabilization of view. The maximum examination time for each eye was 5 min. Images of the cornea (from corneal epithelium to endothelium) and the corneal limbus were collected through moved manually, with each image representing a coronal section of 400 μm × 400 μm.

#### 2.3.2 Image analysis

##### 2.3.2.1 Cornea epithelium

The degree of corneal epithelial deposits in our study was classified according to the grading system proposed by Falke et al. (21), with the images obtained from areas at 12, 3, 6, and 9 o’clock of both the peripheral region (outer third of the cornea, about 1 mm from the limbus) and the central region (a central third of the cornea). A total of 100 images were obtained from each region per eye and images with the maximal number of hyperreflective deposits were chosen for evaluation. A change of two grades in more than 50% of images was interpreted as a grade change in the area as a whole. A grade regression in either the central or the peripheral cornea across 75% of evaluated areas was characterized as a grade change in the cornea overall (21). The grading system of CV according to images acquired by IVCM is listed as follows: In Grade 1, the isolated hyperreflective structures in the basal cell layer account for <25% of cells. When the number of hyperreflective structures in the basal cell layer increases to 25–50% of cells, it is classified as Grade 2. In Grade 3, the pronounced intensity of hyperreflective structures in the basal cell layer accounts for 50–75% of cells. The massive intensity of >75% of cells is classified as Grade 4. Similarly, the grading level of the patient is also assigned as a weighted score (21).

##### 2.3.2.2 Limbus

Situated between the cornea and the sclera, the limbus is categorized into the corneal limbus (CL) and the scleral limbus (SL) (37, 38). The fine, finger-like projections interrupting the regular basal epithelial cells were observed in CL (37, 38). The SL contains the palisades of Vogt (POV), which are fibrovascular structures resembling palisades. These are covered with 2–3 layers of epithelium and alternate with the interpalisadal epithelial rete pegs, containing 10–15 layers of epithelial cells (37, 38). We examined the superior limbus based on previous studies (37, 38). The degree of the limbal deposits was assessed by choosing three frames per location per eye that contained the clearest images of the corneal limbus and scleral limbus areas. We classified the grade of hyperreflective cells in the limbus into three grades (0–2) modified according to the classification of non-pigmented, moderately, and hyperpigmented subjects in previous studies (37, 38).

The grading system of hyperreflective cells in CL and in rete pegs: Grade 0: no hyperreflective cells; Grade 1: moderate hyperreflective cells in the basal epithelial cells, intensity of 0–50%; Grade 2: numerous hyperreflective cells in the basal epithelial cells, intensity of >50%.

The grading system of hyperreflective cells in POV: Grade 0: difficult to distinguish the borders of the basal cells in POV with no hyperreflective basal cells; Grade 2: borders of the basal cells of POV are clear with moderate (0–50%) hyperreflective basal cells; Grade 3: abundant (>50%) adjacent hyperreflective basal cells, making cell borders unrecognizable.

### 2.4 Statistical analysis

Statistical analyses were performed with Statistical Package for the Social Sciences (SPSS) version 27 (IBM Corporation, Armonk, NY, United States). The Shapiro–Wilk test was used to evaluate whether the variables conform with the normal distribution. Continuous variables with normal distribution and ordinal variables were displayed as mean ± standard deviation. Continuous variables with skewed distribution were displayed as median with interquartile range. Independent two-tailed *t*-tests and the Wilcoxon rank–sum test (Mann–Whitney U test) were applied for continuous variables with normal distribution and skewed distribution, respectively. Pearson chi-square test and Fischer’s exact test were applied for the categorical variables as appropriate. Wilcoxon rank sum test (Mann–Whitney U test) and Kruskal–Wallis H test were applied for the ordinal variables as appropriate. McNemar test was applied for the paired nominal variables. Kendall’s tau-b correlation analysis was applied to analyze the correlation of two ordinal variables. Univariate and multivariate logistic regression (ordinal logistic regression) analyses were performed to identify clinical determinants of epithelial deposits in patients with FD. Variables with a significant correlation at the univariable analysis (*p* < 0.05) and several variables (between the sexes and *α*-Gal A activity) have been demonstrated to have a great influence on clinical manifestations in previous studies (2) were further investigated by multivariable models. All *p*-values were two-sided with a statistical significance threshold of 0.05.

## 3 Results

### 3.1 Demographics

The detailed demographic data, including the systemic involvements, duration of the disease, laboratory tests, family history, and accompanying treatments, were presented (Supplementary Tables S1, S2). Sixty eyes from 30 patients with FD (17 males, 13 females; 19 classical phenotypes, 11 non-classical phenotypes) with a mean age of 39.97 ± 14.59 years have been analyzed and compared with 36 eyes from 18 healthy subjects (11 males and 7 females) with a mean age of 41.72 ± 10.32 years. There were no significant differences between the two groups in terms of age (*p* = 0.657) and sex (*p* = 0.762). The mean age was higher in heterozygous females than hemizygous males (47.77 ± 12.76 vs. 34.00 ± 13.29 years, *p* = 0.008) and lower in classical phenotype than non-classical phenotype (35.16 ± 13.89 vs. 48.27 ± 11.60 years, *p* < 0.001).

### 3.2 CV and corneal epithelial deposits

**Figure 1:**
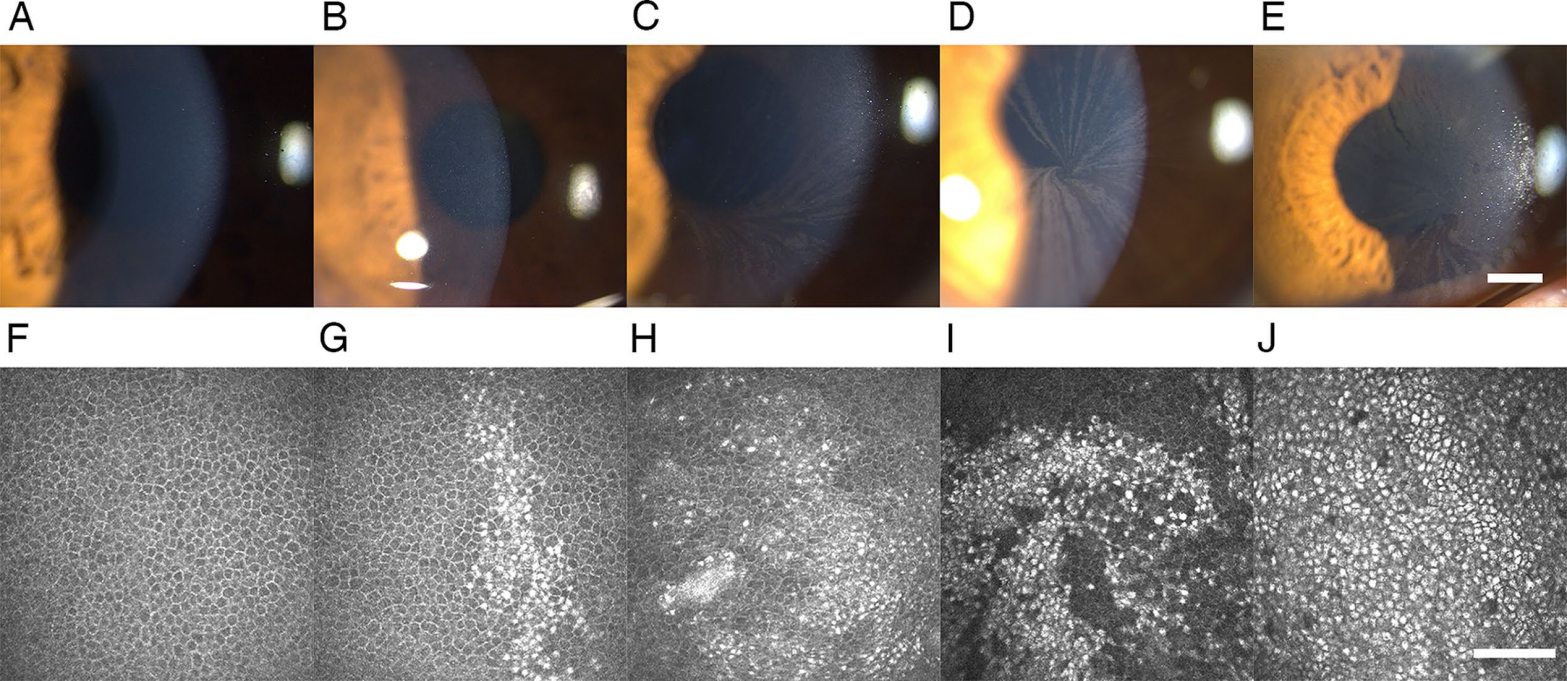
Representative slit-lamp microscopy and IVCM images of CV and epithelial deposits in patients with FD. **(A,F)** Grade 0 CV and epithelial deposits: negative for CV at the slit-lamp microscopy examination and epithelial deposits at the IVCM. **(B,G)** Grade 1 CV and epithelial deposits: several round epithelial microdeposits at the pupillary region of the cornea in the slit-lamp microscopy examination, corresponding to the intracellular hyperreflective pathologic accumulation in the basal cell layer account for <25% of cells in the IVCM. **(C,H)** Grade 2 CV and epithelial deposits: several linear filament-like deposits resembling cat’s whiskers distributed across the boundary of the inferior pupillary margin in the slit-lamp microscopy examination, corresponding to the intracellular hyperreflective pathologic accumulation in the basal cell layer account for 25–50% of cells in the IVCM. **(D,I)** Grade 3 CV and epithelial deposits: the typical whorled pattern of CV extending into the pupillary axis in the slit-lamp microscopy examination, corresponding to the epithelial microdeposits account for 50–75% of cells in the IVCM with morphological distribution of radial stripes producing a whorled pattern at the pupillary axis. **(E,J)** Grade 4 CV and epithelial deposits: clumps of epithelial deposits in the slit-lamp microscopy examination, corresponding to the epithelial microdeposits account for >75% of cells in the IVCM. All slit-lamp microscopy examination images: 25× magnification, bar represents 1 mm. The bar at the IVCM images represents 100 μm. FD, Fabry disease; IVCM, *in vivo* confocal microscopy; CV, cornea verticillata.

At the slit-lamp microscopy examination, the CV was detected in 46 of 60 eyes (76.67%) of patients with FD, with all involved bilaterally (Table 1). CV was typically characterized by cream-colored (ranging from whitish to golden-brown) linear filament-like epithelial deposits extending from the peripheral cornea to the axis of the inferonasal pupillary margin, forming a whorled branching pattern (Figure 1). At the IVCM examination, the corneal epithelial deposits were detected bilaterally in 52 of 60 eyes (86.67%) of patients with FD, manifested as hyperreflective intracellular deposits at the level of basal epithelial cells layer in the cornea (Table 1; Figure 1). All eyes of the healthy controls were negative for CV and corneal epithelial deposits.

**Table 1:** The score of CV, corneal, and limbal deposits grade in heterozygous females and hemizygous males.

	Mean score	Grade 0	Grade 1	Grade 2	Grade 3	Grade 4
CV: Slit-lamp microscopy examination						
Heterozygous (26 eyes)	2.77 ± 1.03	2 (7.69%)	0 (0%)	5 (19.23%)	14 (53.85%)	5 (19.23)
Hemizygous (34 eyes)	1.79 ± 1.49	12 (35.29%)	1 (2.94%)	7 (20.59%)	10 (29.41%)	4 (11.76%)
Fisher’s exact test	*χ*^2^ = 8.410, *p* = 0.056					
Mann–Whitney U test	*Z* = −2.538; *p* = 0.011					
Corneal deposits: IVCM						
Heterozygous (26 eyes)	2.69 ± 0.93	0 (0%)	2 (7.69%)	10 (38.46%)	8 (30.77%)	6 (23.08%)
Hemizygous (34 eyes)	2.62 ± 1.60	8 (23.53%)	0 (0%)	3 (8.82%)	9 (26.47%)	14 (41.18%)
Fisher’s exact test	*χ*^2^ = 16.125, *p* = 0.001					
Mann–Whitney U test	*Z* = −0.697; *p* = 0.486					

	Mean score	Grade 0	Grade 1	Grade 2
Limbal deposits: IVCM				
Corneal limbus				
Heterozygous(26 eyes)	1.77 ± 0.43	0	6 (23.08%)	20 (76.92%)
Hemizygous(34 eyes)	1.88 ± 0.33	0	4 (11.76%)	30 (88.24%)
Pearson chi-square	*χ*^2^ = 1.357, *p* = 0.305			
Mann–Whitney U test	*Z* = −1.155; *p* = 0.305			
Palisades of Vogt				
Heterozygous (26 eyes)	1.31 ± 0.55	1 (3.85%)	16 (61.54%)	9 (34.62%)
Hemizygous (34 eyes)	1.65 ± 0.65	3 (8.82%)	6 (17.65%)	25 (73.53%)
Fisher’s exact test	*χ*^2^ = 12.031, *p* = 0.001			
Mann–Whitney U test	*Z* = −2.569; *p* = 0.009			
Rete pegs				
Heterozygous (26 eyes)	1.23 ± 0.65	3 (11.54%)	14 (53.85%)	9 (34.62%)
Hemizygous (34 eyes)	1.65 ± 0.65	3 (8.82%)	6 (17.65%)	25 (73.53%)
Fisher’s exact test	*χ*^2^ = 9.812, *p* = 0.006			
Mann–Whitney U test	*Z* = −2.702; *p* = 0.007			

IVCM, in vivo confocal microscopy; CV, cornea verticillata.

Through IVCM, we found epithelial deposits not only in all the eyes positive with CV but also in 6 eyes negative with CV, revealing a higher prevalence of epithelial deposits under IVCM (52/60, 86.67%) than the biomicroscopic evaluation of CV under silt lamp (46/60, 76.67%) (*p* = 0.031).

### 3.3 Limbus

**Figure 2:**
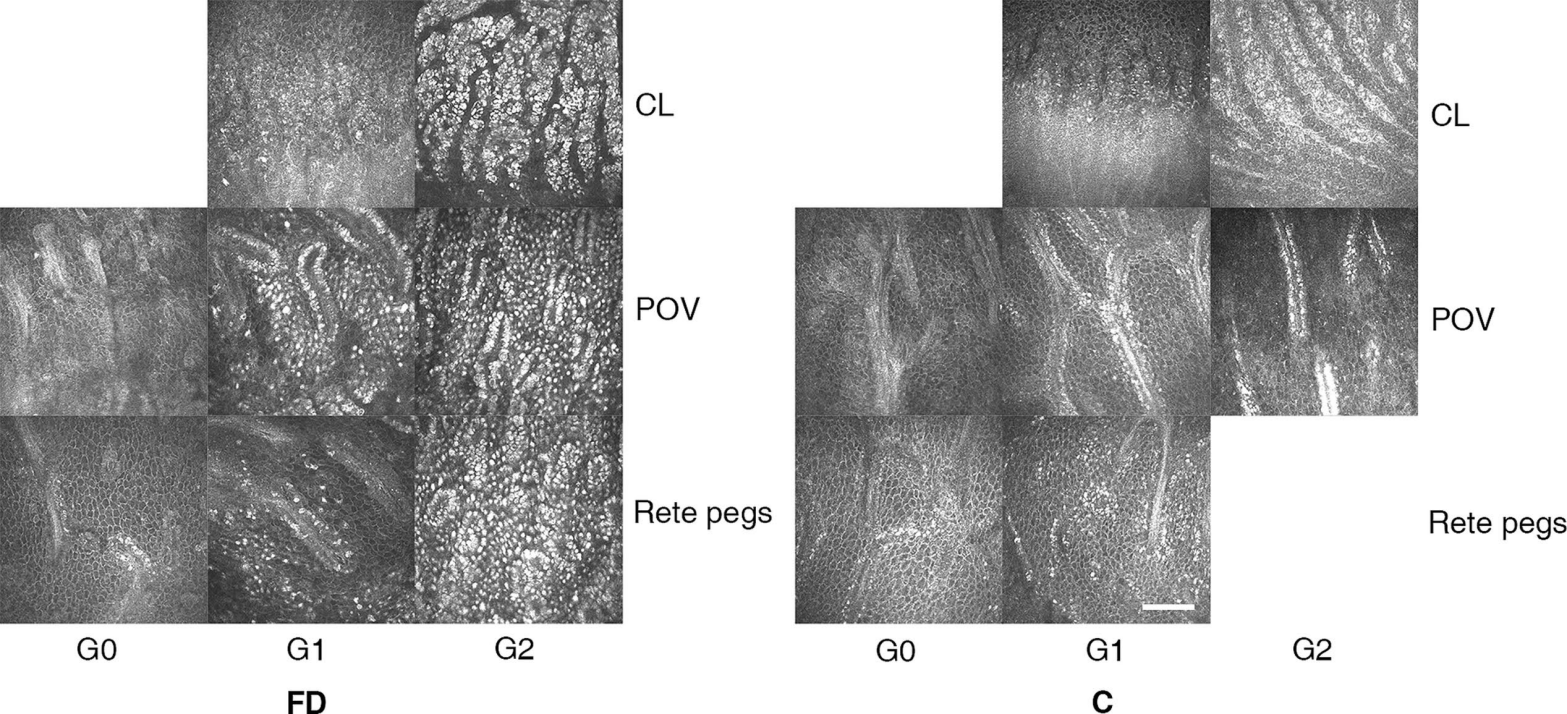
The representative microstructural observations of limbus according to IVCM in patients with FD and healthy controls. In healthy controls, we observed sporadic hyperreflective cells in the CL, POV, and rete pegs, the grade of which quantitatively related to the melanin pigment amount classified into non-pigmented, moderately, and hyperpigmented subjects. In FD, a large account of hyperreflective intracellular deposits at the level of epithelial cells in the limbal region caught great attention. The grading systems of hyperreflective cells in the limbus are listed as follows. CL and in rete pegs: Grade 0: no hyperreflective cells; Grade 1: 0–50% hyperreflective cells; Grade 2: >50% hyperreflective cells. POV: Grade 0: no hyperreflective cells with difficulty in distinguishing the cell borders; Grade 2: moderate (0–50%) hyperreflective cells with clear borders; Grade 3: abundant (>50%) adjacent hyperreflective cells, making cell borders unrecognizable. The bar at the IVCM images represents 100 μm. FD, Fabry disease; C, healthy controls; IVCM, *in vivo* confocal microscopy; CV, cornea verticillata; CL, corneal limbus; POV, palisades of Vogt.

**Figure 3:**
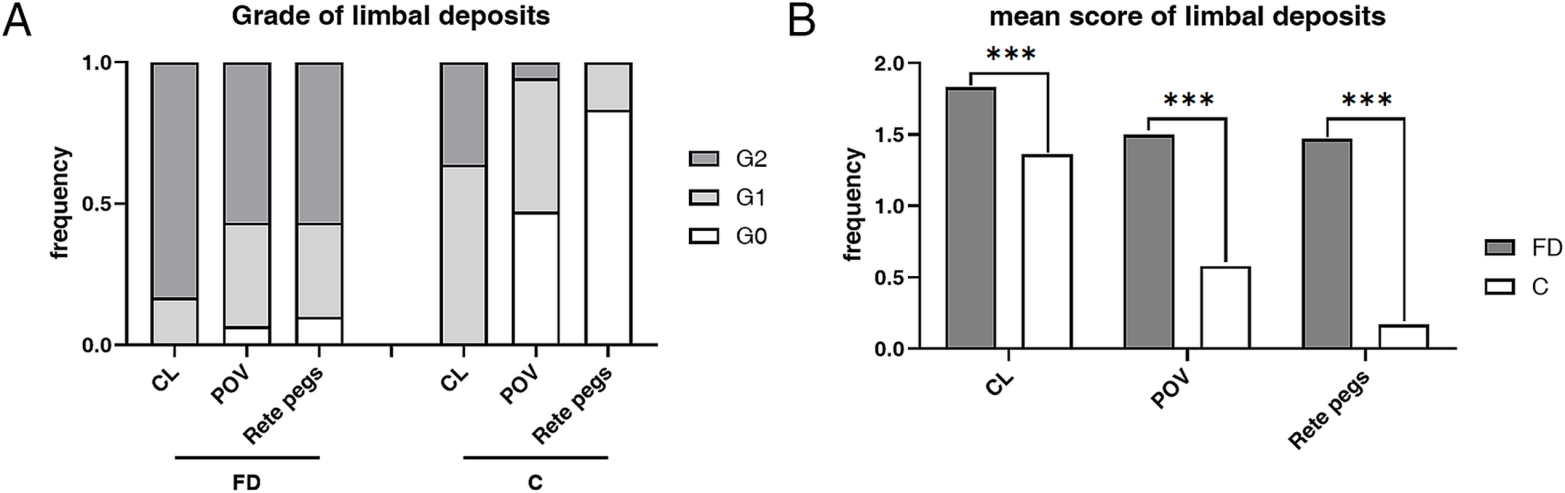
The composition ratio and mean score of grades in limbus hyperrefractive cells according to IVCM examination. **(A)** The composition ratio of different limbal grades in FD and controls. Grade 2 accounted for the majority of patients with FD, while Grades 0 and 1 dominated the controls. **(B)** The weighted average score of limbal grade in FD and controls. The weighted average scores of grades in the CL (*p* < 0.001), POV (*p* < 0.001), and rete pegs (*p* < 0.001) of patients with FD were all statistically higher than those of controls. ***p < 0.001. FD, Fabry disease; C, healthy controls; IVCM, *in vivo* confocal microscopy; CV, cornea verticillata; CL, corneal limbus; POV, palisades of Vogt.

Limbus microstructural observations are presented in Figure 2. The limbus includes the corneal limbus (CL) and scleral limbus (SL), the latter of which also includes palisades of Vogt (POV) and epithelial rete pegs. These hyperreflective cells in the limbus of healthy controls are different amounts of melanin pigment directly related to the skin color, which protects the limbal stem cells from deleterious ultraviolet radiation (36). The prevalence of deposits in POV (93.33% vs. 52.78%, *p* < 0.001) and rete pegs (90.00% vs. 16.67%, *p* < 0.001) were higher in FD than controls (Figure 3). The hyperreflective cells grades in the CL (1.83 ± 0.38 vs. 1.36 ± 0.49, *p* < 0.001), POV (1.50 ± 0.62 vs. 0.58 ± 0.60, *p* < 0.001) and epithelial rete pegs (1.47 ± 0.68 vs. 0.17 ± 0.38, *p* < 0.001) of patients with FD were all statistically higher than that of controls, predominantly focused on Grade 2, attributing to extra accumulation of epithelial deposits in addition to melanin pigment (Figure 3).

In patients with FD, the grade of hyperreflective cells in CL (*r* = 0.453; *p* < 0.001), POV (*r* = 0.588; *p* < 0.001), and epithelial rete pegs (*r* = 0.574; *p* < 0.001) was significantly correlated with the grade of corneal epithelial deposits according to Kendall’s tau-b correlation analysis.

### 3.4 Differences in epithelial deposits between the sexes

**Figure 4:**
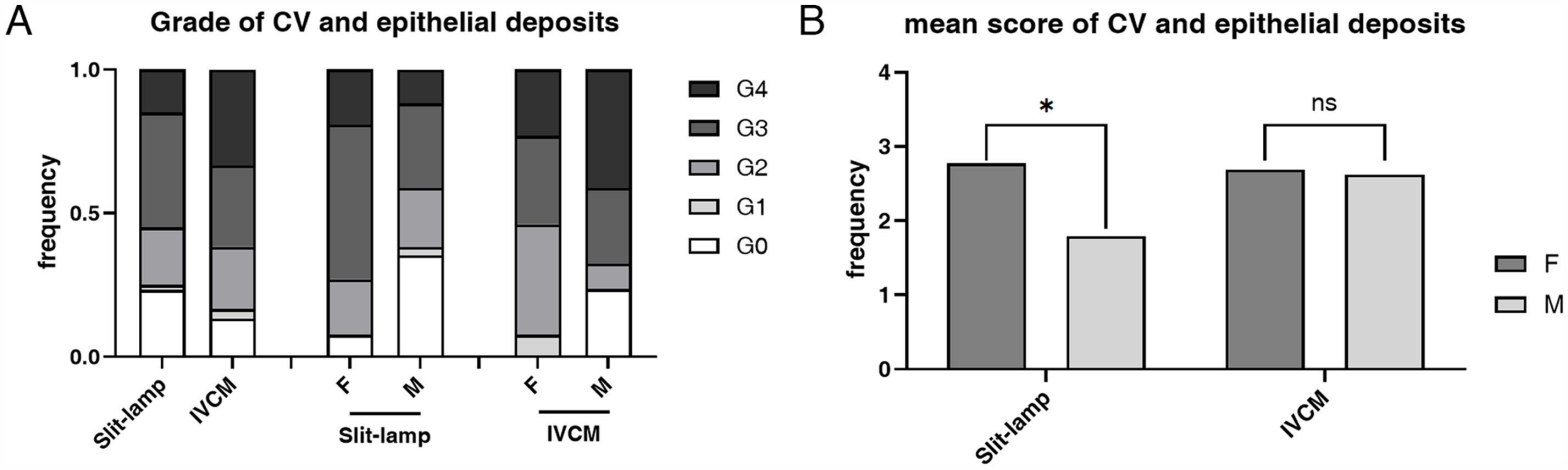
The composition ratio and the mean score of grades of CV according to slit-lamp microscopy examination and epithelial deposits according to IVCM examination. **(A)** The composition ratio of CV and epithelial deposits grades. Although hemizygous individuals typically have more severe overall manifestations, heterozygous individuals have a higher incidence rate of CV (*p* = 0.015) and epithelial deposits (*p* = 0.008). **(B)** The mean score of CV and epithelial deposits Grades. The mean score was higher in the heterozygous individuals than the hemizygous individuals in both slit-lamp microscopy and IVCM examinations, and the difference was statistically significant in slit-lamp microscopy examination (*p* = 0.011). *p < 0.05. F, female; M, male; IVCM, *in vivo* confocal microscopy; CV, cornea verticillata.

The prevalence of both CV (92.31% vs. 64.7%, *p* = 0.015) and epithelial deposits (100.00% vs. 76.47%, *p* = 0.008) was higher in heterozygous females than hemizygous males, respectively (Table 1). The mean score of CV Grades (2.77 ± 1.03 vs. 1.79 ± 1.49, *p* = 0.011) and epithelial deposit grades (2.69 ± 0.93 vs. 2.62 ± 1.60, *p* = 0.486) was higher in heterozygous females than hemizygous males, respectively (Figure 4).

In patients with FD, the prevalence of limbal deposits (*p* > 0.05) did not present a statistical difference between the sexes. The mean score of hyperreflective cells in POV (*p* = 0.009) and epithelial rete pegs (*p* = 0.007) was higher in the hemizygous individuals than the heterozygous individuals (Table 1).

### 3.5 Differences in epithelial deposits among different phenotypes

**Figure 5:**
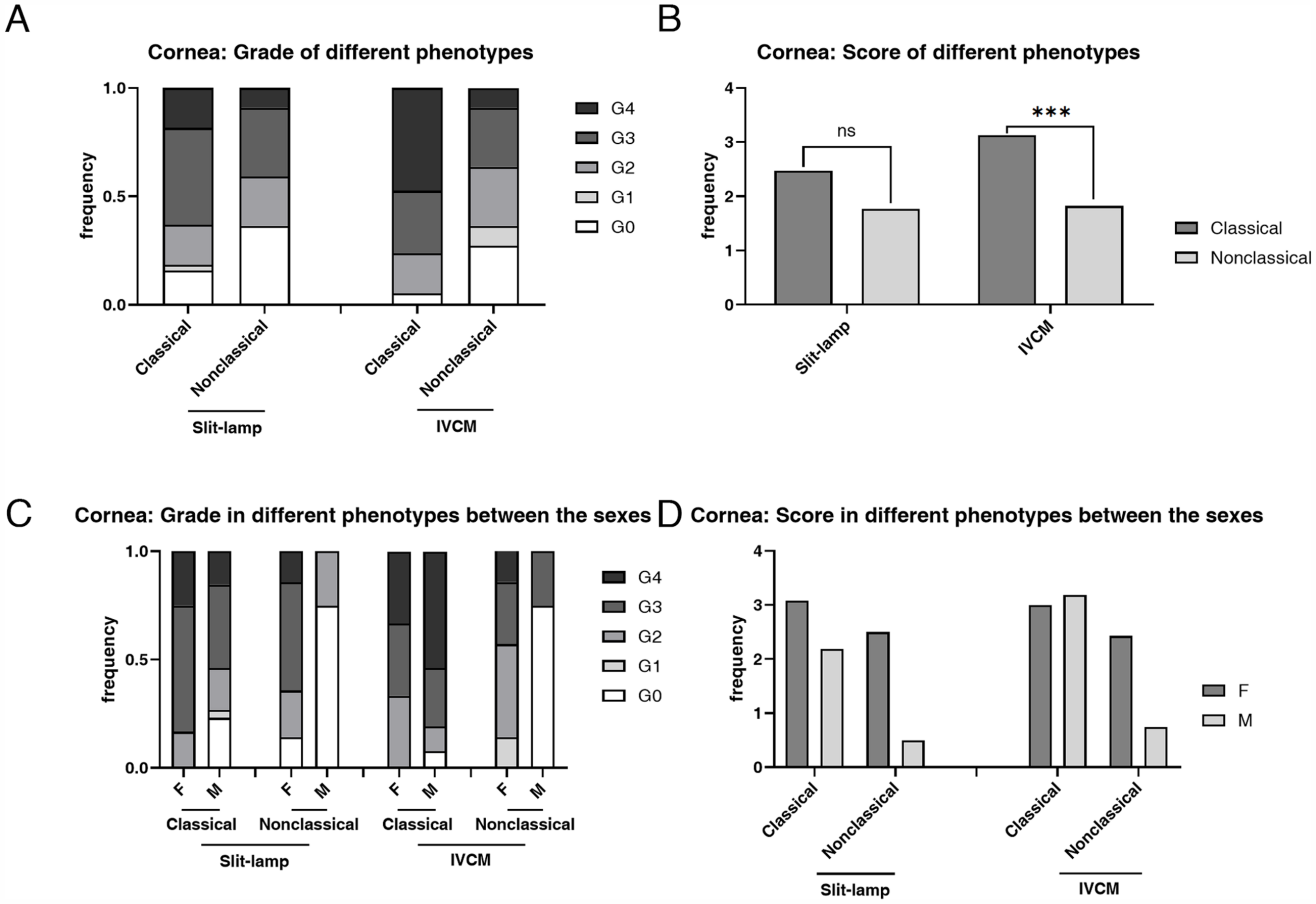
The composition ratio and the mean score of grades of corneal deposits in patients with FD with different phenotypes according to the slit-lamp microscopy and IVCM examinations. **(A)** The composition ratio of grades of CV and corneal hyperrefractive cells in classical and non-classical FD. **(B)** The mean score of grades of CV and corneal hyperrefractive cells in classical and non-classical FD. **(C)** The composition ratio of grades of corneal deposits in different phenotypes between the sexes. **(D)** The mean score of grades of corneal deposits in different phenotypes between the sexes. ***p < 0.001. F, female; M, male; IVCM, *in vivo* confocal microscopy; CV, cornea verticillata.

The prevalence of corneal epithelial deposits was higher in the classical phenotype than in the non-classical phenotype (94.74% vs. 72.73%, *p* = 0.023) (Table 2). The mean score of corneal epithelial deposits grade in the classical phenotype was significantly higher than that in the non-classical phenotype (3.13 ± 1.07 vs. 1.82 ± 1.37, *p* < 0.001) (Table 2; Figure 5). The composition ratio of epithelial deposits grades was significantly different between phenotypes (*p* = 0.002), with Grade 4 taking the largest proportion (47.37%) in the classical group, while Grade 3 (27.27%) and Grade 2 (27.27%) in the non-classical group (Table 2).

**Table 2:** The score of CV and corneal deposits grade in classical and non-classical phenotypes.

	Mean score	Grade 0	Grade 1	Grade 2	Grade 3	Grade 4
Slit-lamp microscopy examination						
Classical phenotype (38 eyes)	2.47 ± 1.29	6 (15.79%)	1 (2.63%)	7 (18.42%)	17 (44.74%)	7 (18.42%)
Non-classical phenotype (22 eyes)	1.77 ± 1.48	8 (36.36%)	0 (0%)	5 (22.73%)	7 (31.82%)	2 (9.09%)
Fisher’s exact test	*χ*^2^ = 4.422, *p* = 0.329					
Mann–Whitney U test	*Z* = −1.847, *p* = 0.065					
Cardiac variant (14 eyes)	1.93 ± 1.33	4 (28.57%)	0 (0%)	3 (21.43%)	7 (50.00%)	0 (0%)
Renal variant (8 eyes)	1.50 ± 1.77	4 (50.00%)	0 (0%)	2 (25.00%)	0 (0%)	2 (25.00%)
Fisher’s exact test	*χ*^2^ = 7.889, *p* = 0.031					
Mann–Whitney U test	*Z* = −0.644, *p* = 0.519					
IVCM						
Classical phenotype (38 eyes)	3.13 ± 1.07	2 (5.26%)	0 (0%)	7 (18.42%)	11 (28.95%)	18 (47.37%)
Non-classical phenotype (22 eyes)	1.82 ± 1.37	6 (27.27%)	2 (9.09%)	6 (27.27%)	6 (27.27%)	2 (9.09%)
Fisher’s exact test	*χ*^2^ = 14.893, *p* = 0.002					
Mann–Whitney U test	*Z* = −3.663, *p* < 0.001					
Cardiac variant (14 eyes)	1.86 ± 1.03	2 (14.29%)	2 (14.29%)	6 (42.86%)	4 (28.57%)	0 (0%)
Renal variant (8 eyes)	1.75 ± 1.91	4 (50.00%)	0 (0%)	0 (0%)	2 (25.00%)	2 (25.00%)
Fisher’s exact test	*χ*^2^ = 9.351, *p* = 0.036					
Mann–Whitney U test	*Z* = 0, *p* = 1.000					

IVCM, in vivo confocal microscopy; CV, cornea verticillata.

**Figure 6:**
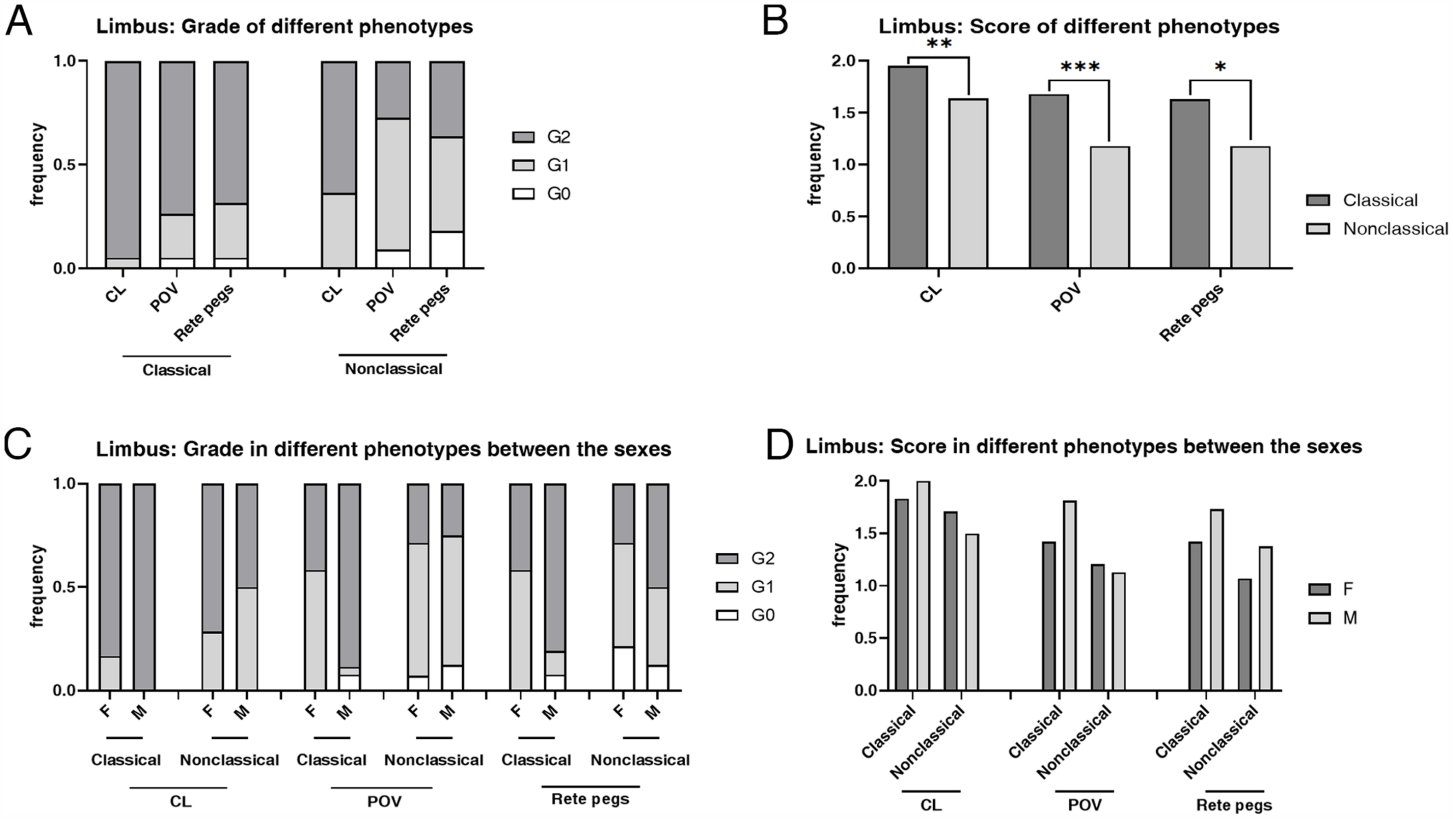
The composition ratio and the mean score of grades in the limbus according to the IVCM examination. **(A)** The composition ratio of limbal grades in classical and non-classical FD. **(B)** The mean score of limbal grades in classical and non-classical FD. **(C)** The composition ratio of limbal grades in different phenotypes between the sexes. **(D)** The mean score of limbal grades in different phenotypes between the sexes. CL, corneal limbus; POV, palisades of Vogt; rete pegs, interpalisadal epithelial rete pegs; F, females; M, males. ***p < 0.001; **p < 0.01; *p < 0.05. F, female; M, male; IVCM, *in vivo* confocal microscopy; CV, cornea verticillata; CL, corneal limbus; POV, palisades of Vogt.

The mean score of hyperreflective cells in CL (*p* = 0.003), POV (*p* < 0.001), and epithelial rete pegs (*p* = 0.014) was statistically higher in the classical phenotype than the non-classical phenotype (Table 3; Figure 6). The prevalence and the mean score of hyperreflective cells in POV (90.91% vs. 52.78%, *p* = 0.003; 1.18 ± 0.59 vs. 0.58 ± 0.60, *p* < 0.001) and rete pegs (81.82% vs. 16.67%, *p* < 0.001; 1.18 ± 0.73 vs. 0.17 ± 0.38, *p* < 0.001) were statistically higher in patients with non-classical FD compared with healthy controls.

**Table 3:** Grades of hyperreflective cells of classical and non-classical phenotypes in the limbus.

	Mean score	Grade 0	Grade 1	Grade 2
Corneal limbus				
Classical phenotype (38 eyes)	1.95 ± 0.226	0 (0%)	2 (5.26%)	36 (94.74%)
Non-classical phenotype (22 eyes)	1.64 ± 0.492	0 (0%)	8 (36.36%)	14 (63.64%)
Pearson chi-square test	*χ*^2^ = 9.703, *p* = 0.003			
Mann–Whitney U test	*Z* = −3.089, *p* = 0.003			
Cardiac variant (14 eyes)	1.57 ± 0.514	0 (0%)	6 (42.86%)	8 (57.14%)
Renal variant (8 eyes)	1.75 ± 0.463	0 (0%)	2 (25.00%)	6 (75.00%)
Pearson chi-square test	*χ*^2^ = 0.702, *p* = 0.649			
Mann–Whitney U test	*Z* = −0.818, *p* = 0.413			
Palisades of Vogt				
Classical phenotype (38 eyes)	1.68 ± 0.574	2 (5.26%)	8 (21.05%)	28 (73.68%)
Non-classical phenotype (22 eyes)	1.18 ± 0.588	2 (9.09%)	14 (63.64%)	6 (27.27%)
Fisher’s exact test	*χ*^2^ = 12.555, *p* < 0.001			
Mann–Whitney U test	*Z* = −3.289, *p* < 0.001			
Cardiac variant (14 eyes)	1.00 ± 0.555	2 (14.29%)	10 (71.43%)	2 (14.29%)
Renal variant (8 eyes)	1.50 ± 0.535	0 (0%)	4 (50.00%)	4 (50.00%)
Fisher’s exact test	*χ*^2^ = 3.336, *p* = 0.244			
Mann–Whitney U test	*Z* = −1.927, *p* = 0.054			
Rete pegs				
Classical phenotype (38 eyes)	1.63 ± 0.589	2 (5.26%)	10 (26.32%)	26 (68.42%)
Non-classical phenotype (22 eyes)	1.18 ± 0.733	4 (18.18%)	10 (45.45%)	8 (36.36%)
Fisher’s exact test	*χ*^2^ = 6.304, *p* = 0.041			
Mann–Whitney U test	*Z* = −2.501, *p* = 0.014			
Cardiac variant (14 eyes)	0.86 ± 0.663	4 (28.57%)	8 (57.14%)	2 (14.29%)
Renal variant (8 eyes)	1.75 ± 0.463	0 (0%)	2 (25.00%)	6 (75.00%)
Fisher’s exact test	*χ*^2^ = 7.647, *p* = 0.030			
Mann–Whitney U test	*Z* = −2.807, *p* = 0.005			

### 3.6 Exploration of correlation in epithelial deposits with other clinical determinants

Supplementary Table S3 illustrates univariate and multivariate logistic regression analyses for a grade of corneal epithelial deposits in patients with FD. After controlling for demographic characteristics, the phenotype (classical and non-classical) (*p* = 0.025) and *α*-Gal A activity (*p* = 0.036) were significantly associated with the grade of corneal epithelial deposits.

In patients with FD, we selected the parameter of epithelial rete pegs grade that differed most significantly between the patient group and healthy subjects for detailed analysis. Supplementary Table S4 illustrates univariate and multivariate logistic regression analyses for a grade of limbal rete pegs epithelial deposits in patients with FD. After controlling for demographic characteristics, sex (*p* = 0.018) was significantly associated with the grade of limbal rete pegs epithelial deposits.

No significant correlation was found between the commencement of systemic treatment (enzyme replacement therapy (ERT) or Venglustat) and the epithelial deposit grade (Supplementary Table S5).

## 4 Discussion

The traditional assessment of CV in clinical practice, until recent times, has primarily been conducted through slit-lamp microscopy. However, the prevalence of CV as observed *via* slit-lamp microscopy has varied across different studies, which may largely depend on the physician’s level of expertise (19). In contrast, IVCM has been proven to be a more precise technique with higher diagnostic power in assessing the presence of corneal epithelial deposits in patients with FD. In a previous comparative study, the presence rate of CV by slit-lamp microscopy examination was 32%, compared with the prevalence of endothelial deposits by IVCM, which was 89%; this indicated a low sensitivity of 36% by slit-lamp microscopy examination (28). In our study, we found that the prevalence of epithelial hyperreflective deposits through IVCM (52/60, 86.67%) was statistically higher than the prevalence of CV through slit-lamp microscopy examination (46/60, 76.67%) (*p* = 0.031), revealing that IVCM has a greater capacity to find mild corneal microdeposits which can easy to be overlooked by the biomicroscopic evaluation. Therefore, the existence of corneal epithelial deposits cannot be excluded through the absence of CV under slit-lamp microscopy examination.

In addition to the corneal deposits, we also evaluated the accumulation of bright intracellular substances at the limbus. As is well known, the corneal epithelial deposits of FD are manifested as hyperreflective intracellular substances, mainly with a whorled branching pattern (15, 16). It is widely accepted that the limbal epithelial stem cells (LESCs) maintain the corneal epithelial renewal, with a centripetal cell migration pathway from the limbus toward the cornea center (30, 39). The hypothesis has been proposed that deposition of undegraded glycosphingolipids accumulate in the LESCs, which differentiate and migrate toward the corneal center and are attributed to the peculiar whorl pattern of keratopathy (31). Herein, we detected the limbus microstructural through IVCM and found the parallel changes of the limbus hyperreflective cells grade with the corneal epithelial deposits grade in patients with FD (*p* < 0.001), consistent with the centripetal movement hypothesis. The centripetal whorl pattern can be visualized in the typical image of IVCM (Figure 1D). In addition, in contrast with healthy controls, the extremely higher incidence and severity of hyperreflective intracellular accumulation in limbus was revealed in the CL, POV, and epithelial rete pegs in particular. This reminds us that in addition to focusing on the cornea, the limbus should also be paid attention to, which is equally important for discovering Gb3 deposit accumulation.

FD is an X-linked recessive hereditary condition, in which females are heterozygous and males are hemizygous, leading to phenotypic variations in the clinical manifestations of the disease (40–42). Heterozygous females have a longer average lifespan, reaching 55.4–70 years (40, 42). The heterozygous and hemizygous are shorter than that of the general population by 20–25 years and 15–30 years, respectively (40–42). Among various disorders, the main cause of death in hemizygous males is renal failure, accounting for up to 54.5%, while heterozygous females mainly succumb to cerebrovascular accidents and cardiac complications (40–42). In this study, by slit-lamp microscopy examination and corneal confocal microscopy, we also observed sex differences in phenotypic manifestations.

Heterozygous females exhibited heavier corneal phenotypes. For instance, among heterozygous females, only 2/26 (7.69%) had no CV with a mean CV score of 2.77 ± 1.03, while 12/34 (35.29%) in hemizygous males with a mean score of 1.79 ± 1.49. Previous studies have described the asymmetrical prevalence of CV between the sexes while drawing different conclusions about hemizygotes or heterozygotes having a higher prevalence (12, 16, 19). Evidently, in our study, the corneal phenotypic manifestations of heterozygous females were significantly more severe than those of hemizygous males, implying a higher level of corneal glycosphingolipid accumulation, which may result from a delayed diagnosis and longer duration of the disease, despite their residual enzymatic activity.

In contrast to corneal phenotypes, hemizygous males showed more pronounced limbus phenotypes. For example, among hemizygous males, 25/34 (73.53%) exhibited Grade 2 rete pegs, compared to 9/26 (34.62%) in heterozygous females. Consistent with the changes observed in systemic manifestations, the manifestations at the limbus also reveal a higher grade of hyperrefractive cells in the scleral limbus of the hemizygous males (4, 41–43). Attributed to the random inactivation of one X chromosome in females, called lyonization, heterozygous females are mosaic for the expression of *α*-GalA, which leads to more residual enzyme activity and mild characteristic FD symptoms (4, 8, 41–45). The limbus is where the LESCs exist, where the undegraded Gb3 within the lysosomes accumulate initially with a heavier pattern in males, resulting in the accumulation of hyperreflective intracellular deposits observed through IVCM. With the migration from limbus toward the central cornea, the LESCs with Gb3 divide and produce transient amplifying cells to move centripetally until they reach the corneal epithelium as terminally differentiated cells with Gb3 (30). In this study, although less Gb3 was found in the limbus of heterozygous females, the Gb3 in the corneal epithelium may accumulate more through the corneal epithelial maintenance from LESCs with a longer duration of the disease, thus explaining why the corneal phenotypic manifestations were found more severe in heterozygous females.

The two phenotypes of FD, early-onset classical FD, and late-onset non-classical FD, have pleiotropic form and different severity of manifestations in multiple organ systems (4, 46). In this study of ocular manifestations, we observed phenotypic differences as well. According to the IVCM, the classical FD exhibited heavier corneal and limbus manifestations. For instance, the prevalence of corneal epithelial deposits at IVCM was statistically higher in the classical phenotype (94.74%) than the non-classical phenotype (72.73%) (*p* = 0.023). A greater account of Grade 4 epithelial deposits and higher mean score of deposits in grades were observed in classical FD compared to non-classical FD. This trend of phenotypic difference was also proven in the manifestation of limbus deposits. The classical FD, especially classical hemizygous males, exhibited a higher mean score of hyperreflective cells in the limbus, in accordance with the known phenomenon of higher events risk in males with classical FD than both males with non-classical FD and females (4). Although nonclassical FD often presents with milder clinical symptoms, this study found that the prevalence and mean score of hyperreflective cells in the POV and rete pegs of limbus were statistically higher in non-classical FD patients than in healthy controls. Moreover, in the non-classical phenotype, the prevalence of hyperreflective cells in POV (90.91%) and rete pegs (81.82%) was even higher than that in the corneal epithelium (72.73%). The detection of limbus plays a complementary role in improving the detection rate in patients without characteristic manifestations, especially those with negative CV.

After adjusting demographic characteristics, the phenotypes remain independently associated with the grade of corneal epithelial deposits, supporting the concept of more severe corneal deposits in patients with classical FD. Confounding factors, including manifestations of peripheral nerve, skin and gastrointestinal (GI), and duration of the disease had no significant effect on the outcome of epithelial deposits grade. No significant correlation was found between the commencement of systemic treatment with the epithelial deposits grade in our study. However, due to a lack of cohort data, it is unclear whether the degree of corneal deposition will be altered during treatment, which needs to be further explored in the future studies. It is worth mentioning that a high prevalence of corneal and limbus deposits was identified in two variants of the nonclassical phenotype, especially in the cardiac variant. In the cardiac variant, the prevalence of CV and corneal hyperreflective cells was 71.43 and 85.71%, respectively. In the renal variant, the prevalence of CV and corneal hyperreflective cells was both 50.00%. According to previous studies and guidelines, it was previously thought that the non-classical phenotype, especially the cardiac variant, often lacks classic manifestations of CV (33, 47, 48). In contrast to the previous studies suggesting no characteristic features of CV in cardiac variants (35), our study found positive CV *via* slit-lamp microscopy examination and corneal hyperreflective cells in the IVCM. This reminds us of the variable, non-negligible ocular manifestations in cardiac variants, which need to be confirmed in a larger sample size in the future.

A limitation of this study is its small sample size. Especially in the prevalence of CV, patients with a classical phenotype have shown a higher prevalence than those with a non-classical phenotype, although without statistical significance, which necessitates us to accumulate more cases and expand the sample size to further verify whether this result is correct. As a study of a rare disease, we included data from both eyes of the patients in the study, which may have introduced a certain degree of bias to the results. Moreover, after grouping patients by sex and disease type, the percentage of patients in each group decreased even smaller. The small sample may lead to bias in the multifactor regression analysis used to control for potential confounders. Including the real-world patient population in a specialized center is a strength of this study. To further validate the findings of this study, we plan to spend more time collecting data to increase the sample size.

This study summarized the prevalence and severity of epithelial deposits in both cornea and limbus of patients with FD, and correlated these findings with sex and phenotypic variations. We hope the IVCM will play an essential role in the early diagnosis of FD in the future.

## Data Availability

The original contributions presented in the study are included in the article/Supplementary material, further inquiries can be directed to the corresponding author.
