# Supplementary material for: In vivo confocal microscopic study of cornea verticillata and limbus deposits in patients with Fabry disease
Source: Front Med (Lausanne). 2025 Feb 5;12:1541510. doi: 10.3389/fmed.2025.1541510 (PMC11836033; doi:10.3389/fmed.2025.1541510)
Supplement: Supplementary file 1 [file Table_1.DOCX]

**Supplementary table 1.** The detailed demographic data of each patient with FD. FD: fabry disease; P: patient; α-Gal A: α-Galactosidase A; Lyso-Gb3: globotriaosylsphingosine; CHD: coronary heart disease; GI: gastrointestinal; CVA: cerebralvascular accident; HCM: hypertrophic cardiomyopathy; LVH: left ventricular hypertrophy; CKD: chronic kidney disease.

| P | Age | Gender | Onset age | Systemic manifestations | α-Gal A activity (μmol/L/h) | Lyso-Gb3 (ng/mL) | GLA variants | Phenotype | Treatment/  duration | Family history |
| --- | --- | --- | --- | --- | --- | --- | --- | --- | --- | --- |
| P1 | 40 | M | 20 | Neuropathic pain; tinnitus; nausea and dizziness, sudden deafness; proteinuria; CHD | 0.2 | 200 | exon2 deletion | Classical | agalsidase-β, 5 years; venglustat 1years | Mother |
| P2 | 24 | F | 10 | Neuropathic pain; tinnitus; angiokeratoma | 1.61 | 5.09 | c.101A>G, p.Asn34Ser | Classical | Gabapentin | None |
| P3 | 21 | M | 5 | Neuropathic pain; hypohidrosis; ankle swelling; angiokeratoma; GI symptoms; | 0.42 | 98.69 | c.1196C>A, p.Trp399* | Classical | Venglustat, 2 years | elder male cousin |
| P4 | 40 | M | 18 | Neuropathic pain; CVA; renal transplant; angiokeratoma; HCM | 0.32 | 97.3 | c.929T>G, p.Leu310Arg | Classical | agalsidase-β, 2 years | Mother (P5); |
| P5 | 64 | F | 63 | dizziness, Tinnitus; atrial fibrillation | 1.93 | 3.91 | c.929T>G, p.Leu310Arg | Nonclassical, cardiac variant | None | Son (P4) |
| P6 | 50 | F | 10 | Neuropathic pain, hypohidrosis; CVA; proteinuria; chest distress, breath obstruction; HCM | 3.64 | 12.51 | c.266T>G, p.Leu89Arg | Classical | agalsidase-β, 1 years; agalsidase-α, 0.5 years | None |
| P7 | 50 | F | 40 | Dizziness; proteinuria; left ventricular hypertrophy; hypertension; breath obstruction | 26 | 15.4 | c.658C>T. p.(Arg220*) | Nonclassical, cardiac variant | None | Son, sister, nephew |
| P8 | 34 | F | 32 | Proteinuria; GI symptoms | 16.5 | 1.43 | c.G511C, p.Gly171Arg | Nonclassical, renal variant | None | Father |
| P9 | 58 | F | 50 | Tinnitus, dizziness; GI symptoms; chest pain, palpitation | 3.11 | 1.17 | c.874G>C, p.Ala292Pro | Nonclassical, cardiac variant | None | Son; Sister (P10) |
| P10 | 60 | F | 7 | Neuropathic pain, hypohidrosis; dizziness; ankle swelling; HCM; breath obstruction | - | - | c.874G>C, p.Ala292Pro | Classical | None | Sister (P9); nephew |
| P11 | 51 | M | 6 | Neuropathic pain; hypohidrosis; proteinuria; angiokeratoma; GI symptoms | 0.33 | 136 | c.738_775del | Classical | None | None |
| P12 | 58 | F | 48 | Hypohidrosis; atrial fibrillation; HCM | 2.07 | 5.2 | c.440G>A, p.Gly147Glu | Nonclassical, cardiac variant | None | Son |
| P13 | 35 | M | 5 | Neuropathic pain; hypohidrosis; sensorineural hearing loss; CVA; ankle swelling; renal dialysis; GI symptoms; HCM | 2.4 | 18.19 | c.453C>A, p.Tyr151* | Classical | agalsidase-α, 6years | Mother; sister |
| P14 | 14 | M | 8 | Tinnitus; Neuropathic pain | 0.4 | 87.84 | c.1008delC, p.Phe337fs | Classical | carbamazepine | none |
| P15 | 41 | M | 30 | Proteinuria; abnormal renal function | 0.51 | 2.73 | c.506T>C, p.Phe169Ser | Nonclassical, renal variant | agalsidase-α, 0.5 years | Mother; two uncles |
| P16 | 17 | M | 16 | Neuropathic pain | 0.33 | 1.46 | c.335G>A, p.(Arg112His) | Classical | None | Mother |
| P17 | 52 | M | 46 | hypohidrosis; deafness; HCM, sick sinus syndrome; chest distress, breath obstruction; heart failure, pacemaker implantation; ankle swelling; proteinuria | 0.33 | 94.66 | c.101A>G, p.Asn34Ser | Classical | None | Mother |
| P18 | 18 | M | 8 | Neuropathic pain; hypohidrosis; angiokeratoma; hypertension | 0.35 | 101.45 | c.146 G>T, p.(Arg49Leu) | Classical | None | elder female cousin (P19); maternal aunt (P30) |
| P19 | 32 | M | 8 | Hypohidrosis; neuropathic pain; tinnitus, hearing loss, dizziness, rotating vision; proteinuria; angiokeratoma; GI symptoms | 0.73 | 80.79 | c.146 G>T, p.(Arg49Leu) | Classical | Venglustat, 1years | Mother (P30); younger male cousin (P18) |
| P20 | 32 | F | 24 | GI symptoms; palpitation | 3.31 | 3.12 | c.680G>A, p.Arg227Gln | Nonclassical, cardiac variant | None | Father; father's sister and brother |
| P21 | 30 | M | 8 | Neuropathic pain; hypohidrosis; proteinuria; angiokeratoma; GI symptoms; hypertension; palpitation; | 0.5 | 127 | exon 6 duplication | Classical | Venglustat, 0.5years | None |
| P22 | 21 | M | 12 | Neuropathic pain; hypohidrosis; tinnitus; angiokeratoma; GI symptoms; hypertension | 0.2 | 97.3 | c.511G>T, p.Gly171Cys | Classical | None | Mother |
| P23 | 29 | M | 7 | Neuropathic pain; hypohidrosis; tinnitus, dizziness; angiokeratoma; GI symptoms; chest distress, palpitation | 0.5 | 53.55 | exon3 c.503A>G, p.Lys168Arg | Classical | None | Mother; daughter |
| P24 | 33 | F | 8 | Neuropathic pain | 0.57 | 8.45 | GLA c.877C>T, p.Pro293Ser | Classical | None | Two sons |
| P25 | 61 | M | 50 | Proteinuria; HCM | 0.23 | 7.2 | c.717A>G, p.Ile239Met | Nonclassical, cardiac variant | None | None |
| P26 | 57 | F | 47 | Palpitation; CHD; HCM | 1.42 | 3.65 | c.334C>T, p.Arg112Cys | Nonclassical,  cardiac variant | None | Son |
| P27 | 39 | M | 8 | Neuropathic pain; Tinnitus; chronic kidney diseases; GI symptoms; LVH | 2.31 | 105.69 | c.1024C>T, p.Arg342* | Nonclassical, renal variant | agalsidase-α, 1years | daughter |
| P28 | 50 | F | 10 | Neuropathic pain; Proteinuria; HCM; breath obstruction | 0.9 | 51.71 | c.463G>C, p.Asp155His | Classical | agalsidase-α, 0.25 years | brother |
| P29 | 37 | M | 30 | Proteinuria; CKD stage 4 | 1.93 | 20.31 | c.561G>A, p.Met187Ile | Nonclassical, renal variant | agalsidase-α, 1year | none |
| P30 | 51 | F | 5 | Neuropathic pain; HCM | 0.51 | 6.79 | c.146 G>T, p.(Arg49Leu) | Classical | None | Daughter (P19); nephew (P18) |
