# Supplementary material for: In vivo confocal microscopic study of cornea verticillata and limbus deposits in patients with Fabry disease
Source: Front Med (Lausanne). 2025 Feb 5;12:1541510. doi: 10.3389/fmed.2025.1541510 (PMC11836033; doi:10.3389/fmed.2025.1541510)
Supplement: Supplementary file 2 [file Table_2.DOCX]

**Supplementary table 2.** The general demographic data of patients with FD. FD: fabry disease; IQR: interquartile range; M: male; F: female; α-Gal A: α-Galactosidase A; Lyso-Gb3: globotriaosylsphingosine; CHD: coronary heart disease; GI: gastrointestinal; TIA: transient ischemic attack; CVA: cerebralvascular accident; HCM: hypertrophic cardiomyopathy; LVH: left ventricular hypertrophy; CKD: chronic kidney disease.

| Variables | | Total sample (n=30)  N (%)/ median (range, IQR) |
| --- | --- | --- |
| Sex (M/F) | | 17/13 |
| Duration of disease | | 11.00 (1.00-46.00, 16.25) |
| α-Gal A activity (μmol/L/h) (n=29) | | 0.57 (0.20-26.00, 1.79) |
| Lyso-Gb3 (ng/mL) (n=29) | | 18.19 (1.17-200.00, 92.51) |
| Initiation of ERT or Venglustat | | 11 (36.67) |
| Peripheral nerve manifestations | | 24 (80.00) |
|  | Neuropathic pain | 19 (63.33) |
|  | Hypohidrosis | 12 (40.00) |
|  | Tinnitus | 8 (26.67) |
|  | Hearing loss | 5 (16.67) |
|  | Nausea and dizziness | 7 (23.33) |
| Cerebrovascular manifestations: TIA or CVA | | 3 (10.00) |
| Renal manifestations | | 14 (46.67) |
|  | Ankle swelling | 4 (13.33) |
|  | Proteinuria | 14 (46.67) |
|  | Renal insufficiency | 5 (16.67) |
|  | End stage renal failure, renal dialysis or renal transplant | 3 (10.00) |
| Skin manifestation: angiokeratoma | | 8 (26.67) |
| GI manifestations | | 11 (36.67) |
| Cardiovascular manifestations | | 20 (66.67) |
|  | Chest pain, chest distress, breath obstruction, palpitation, atrial fibrillation | 11 (36.67) |
|  | LVH or HCM | 12 (40.00) |
|  | CHD | 2 (6.67) |
|  | Hypertension | 3 (10.00) |
