# Supplementary material for: In vivo confocal microscopic study of cornea verticillata and limbus deposits in patients with Fabry disease
Source: Front Med (Lausanne). 2025 Feb 5;12:1541510. doi: 10.3389/fmed.2025.1541510 (PMC11836033; doi:10.3389/fmed.2025.1541510)
Supplement: Supplementary file 3 [file Table_3.DOCX]

**Supplementary table 3.** Univariate and multivariate logistic regression analyses for grade of corneal epithelial deposits in the patients with FD. FD: fabry disease; α-Gal A: α-Galactosidase A; GI: gastrointestinal; ERT: enzyme replacement therapy.

|  | Univariable | | Multivariable | |
| --- | --- | --- | --- | --- |
|  | Beta | *p-*value | Beta | *p-*value |
| Phenotype | 1.951 | < 0.001 | 2.012 | 0.025 |
| Gender | -0.329 | 0.484 | 0.420 | 0.497 |
| Peripheral nerve manifestations | -2.462 | < 0.001 | -0.990 | 0.234 |
| Cerebrovascular manifestations | -1.773 | 0.055 | - | - |
| Renal manifestations | 0.363 | 0.437 | - | - |
| Skin manifestation**:** angiokeratoma | -1.696 | 0.004 | -0.657 | 0.389 |
| GI manifestations | -1.006 | 0.045 | -1.146 | 0.071 |
| Cardiovascular manifestations | -0.128 | 0.794 | - | - |
| Duration of disease | 0.036 | 0.039 | 0.005 | 0.824 |
| α-Gal A activity | 0.028 | 0.535 | 0.119 | 0.036 |
| ERT or Venglustat | 0.316 | 0.428 | - | - |
