# Supplementary material for: In vivo confocal microscopic study of cornea verticillata and limbus deposits in patients with Fabry disease
Source: Front Med (Lausanne). 2025 Feb 5;12:1541510. doi: 10.3389/fmed.2025.1541510 (PMC11836033; doi:10.3389/fmed.2025.1541510)
Supplement: Supplementary file 4 [file Table_4.DOCX]

**Supplementary table 4.** Univariate and multivariate logistic regression analyses for grade of limbal rete pegs epithelial deposits in the patients with FD. FD: fabry disease; α-Gal A: α-Galactosidase A; GI: gastrointestinal; ERT: enzyme replacement therapy.

|  | Univariable | | Multivariable | |
| --- | --- | --- | --- | --- |
|  | Beta | *p-*value | Beta | *p-*value |
| Phenotype | 1.342 | 0.013 | 1.564 | 0.117 |
| Gender | -1.436 | 0.008 | -1.730 | 0.018 |
| Peripheral nerve manifestations | -1.328 | 0.035 | -0.677 | 0.498 |
| Cerebrovascular manifestations | -0.577 | 0.526 | - | - |
| Renal manifestations | -0.705 | 0.175 | - | - |
| Skin manifestation**:** angiokeratoma | -2.997 | 0.006 | -1.696 | 0.170 |
| GI manifestations | -1.889 | 0.003 | -1.533 | 0.118 |
| Cardiovascular manifestations | 1.064 | 0.071 | - | - |
| Duration of disease | 0.016 | 0.393 | - | - |
| α-Gal A activity | 0.085 | 0.261 | 0.292 | 0.112 |
| ERT or Venglustat | -0.645 | 0.236 | - | - |
