# Supplementary material for: In vivo confocal microscopic study of cornea verticillata and limbus deposits in patients with Fabry disease
Source: Front Med (Lausanne). 2025 Feb 5;12:1541510. doi: 10.3389/fmed.2025.1541510 (PMC11836033; doi:10.3389/fmed.2025.1541510)
Supplement: Supplementary file 5 [file Table_5.DOCX]

**Supplementary table 5.** Univariate and multivariate logistic regression analyses for grade of corneal and limbal epithelial deposits in the patients with FD. FD: fabry disease; ERT: enzyme replacement therapy.

|  | Univariable | | Multivariable | |
| --- | --- | --- | --- | --- |
|  | Beta | *p-*value | Beta | *p-*value |
| Corneal deposits | | | | |
| Phenotype | 1.951 | < 0.001 | 2.221 | < 0.001 |
| ERT or Venglustat | 0.316 | 0.513 | 0.902 | 0.077 |
| Corneal limbus | | | | |
| Phenotype | 2.331 | 0.006 | 2.464 | 0.005 |
| ERT or Venglustat | 0.170 | 0.811 | 0.648 | 0.425 |
| Palisades of Vogt | | | | |
| Phenotype | 1.805 | 0.002 | 1.759 | 0.002 |
| ERT or Venglustat | -0.582 | 0.285 | -0.371 | 0.525 |
| Rete pegs | | | | |
| Phenotype | 1.342 | 0.013 | 1.277 | 0.019 |
| ERT or Venglustat | -0.645 | 0.236 | -0.478 | 0.396 |
| Corneal deposits | | | | |
| Gender | -0.329 | 0.484 | -0.536 | 0.297 |
| ERT or Venglustat | 0.316 | 0.513 | 0.534 | 0.312 |
| Corneal limbus | | | | |
| Gender | -0.811 | 0.251 | -1.074 | 0.180 |
| ERT or Venglustat | 0.170 | 0.811 | 0.635 | 0.435 |
| Palisades of Vogt | | | | |
| Gender | -1.368 | 0.012 | -1.361 | 0.021 |
| ERT or Venglustat | -0.582 | 0.285 | -0.018 | 0.977 |
| Rete pegs | | | | |
| Gender | -1.436 | 0.008 | -1.396 | 0.017 |
| ERT or Venglustat | -0.645 | 0.236 | -0.106 | 0.862 |
